# Vitamin D therapy in chronic kidney disease: a critical appraisal of clinical trial evidence

**DOI:** 10.1093/ckj/sfae227

**Published:** 2024-07-18

**Authors:** Wing-Chi G Yeung, Nigel D Toussaint, Sunil V Badve

**Affiliations:** Department of Nephrology, Wollongong Hospital, Wollongong, New South Wales, Australia; Renal and Metabolic Division, The George Institute for Global Health, Sydney, New South Wales, Australia; Faculty of Medicine, University of New South Wales, Sydney, New South Wales, Australia; Department of Nephrology, The Royal Melbourne Hospital, Parkville, Victoria, Australia; Department of Medicine (RMH), University of Melbourne, Parkville, Victoria, Australia; Renal and Metabolic Division, The George Institute for Global Health, Sydney, New South Wales, Australia; Faculty of Medicine, University of New South Wales, Sydney, New South Wales, Australia; Department of Nephrology, St George Hospital, Sydney, New South Wales, Australia

**Keywords:** bone health, cardiovascular disease, chronic kidney disease, CKD-MBD, vitamin D

## Abstract

In people with chronic kidney disease (CKD), the physiology of vitamin D is altered and leads to abnormalities in bone and mineral metabolism which contribute to CKD mineral and bone disorder (CKD-MBD). Observational studies show an association between vitamin D deficiency and increased risk of mortality, cardiovascular disease and fracture in CKD. Although vitamin D therapy is widely prescribed in people with CKD, clinical trials to date have failed to demonstrate a clear benefit of either nutritional vitamin D supplementation or active vitamin D therapy in improving clinical outcomes in CKD. This review provides an updated critical analysis of recent trial evidence on vitamin D therapy in people with CKD.

## INTRODUCTION

Chronic kidney disease mineral and bone disorder (CKD-MBD) is a common complication in people with CKD, characterized by abnormalities in mineral and bone metabolism and vascular calcification. Changes in vitamin D metabolism in CKD are thought to play an important role in the pathogenesis of CKD-MBD. CKD-MBD has been linked to increased risk of fractures, bone pain, cardiovascular disease and mortality in people with CKD. Cardiovascular disease is the leading cause of death in this population, with excess cardiovascular risk related to factors including vascular calcification, arterial stiffness, left ventricular hypertrophy and endothelial dysfunction.

An important and common treatment of CKD-MBD for many years has been vitamin D therapy. Vitamin D compounds can be classified as nutritional or active, depending on whether they have any direct action on the vitamin D receptor. Active compounds are also commonly referred to as vitamin D receptor activators (VDRAs). Calcitriol is the naturally occurring VDRA, but many synthetic analogues are available including alfacalcidol, maxacalcitol, paricalcitol and doxercalciferol. The most recent Kidney Disease: Improving Global Outcomes (KDIGO) CKD-MBD guidelines published in 2017 suggest that vitamin D deficiency and insufficiency should be treated in the same way as the general population (2C recommendation) [1]. The use of calcitriol and VDRAs should be reserved for people with CKD stages 4 and 5 with severe hyperparathyroidism (ungraded), though the optimal parathyroid hormone (PTH) target is not known in people not on dialysis.

Over a hundred trials have been performed on vitamin D therapy in CKD, but the majority have focused on biochemical outcomes. A 2023 systematic review found moderate quality evidence that VDRAs effectively suppress PTH levels, but at the expense of increased risk of hypercalcaemia [2]. There is still ongoing uncertainty about the benefits of vitamin D therapy on clinical outcomes. This narrative review will provide a critical appraisal of current trial evidence on mortality, cardiovascular disease and bone outcomes in people with CKD.

## ASSOCIATION BETWEEN VITAMIN D DEFICIENCY AND ADVERSE OUTCOMES IN CKD

**Figure 1:**
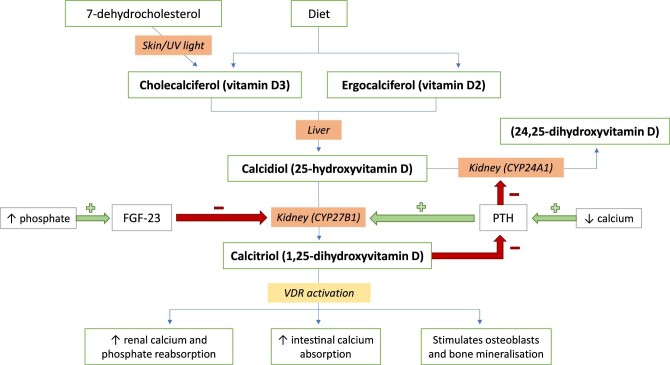
Normal vitamin D metabolism and function.

Under normal physiological conditions, vitamin D2 and D3 are synthesized in the human skin or ingested through dietary intake. They go through sequential hydroxylation, first in the liver to 25-hydroxyvitamin D [25(OH)D], then to its biologically active form, 1,25-dihydroxyvitamin D [1,25(OH)_2_D] in the kidneys (Fig. 1). The classical function of vitamin D is to maintain bone health by regulating calcium homeostasis and mineral metabolism [3]. In states of vitamin D deficiency, PTH production is upregulated, which leads to increased bone turnover and bone resorption. In children, this can present as rickets. In CKD, 1,25(OH)_2_D deficiency and progressive hyperphosphataemia lead to the development of secondary hyperparathyroidism and CKD-MBD.

**Figure 2:**
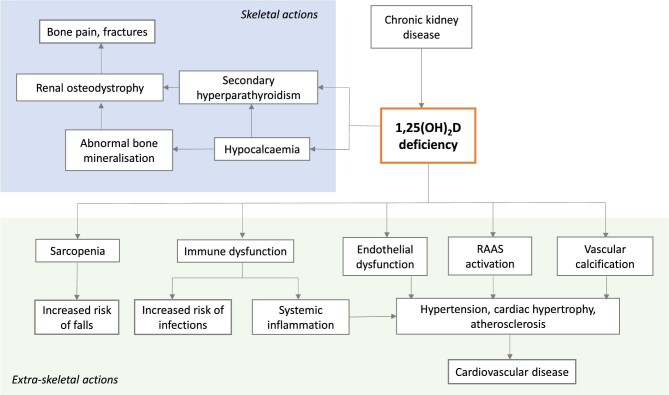
Proposed systemic effects of vitamin D deficiency in CKD.

Over the years, the ‘extra-skeletal’ effects of vitamin D on muscle, cardiovascular, nervous and immune functions have also been studied (Fig. 2). There are several proposed mechanisms for how vitamin D may affect cardiovascular health, including effects on endothelial function [4–6], the renin–angiotensin–aldosterone system [7], blood pressure [8], inflammation [9] and vascular calcification [10–12]. Vascular calcification causes stiffening of major arteries, thereby increasing left ventricular afterload, and may contribute to the development of left ventricular hypertrophy in people with CKD [13, 14]. The degree of vascular calcification strongly predicts cardiovascular and all-cause mortality [15, 16].

Serum 25(OH)D levels are routinely used as a marker of vitamin D sufficiency, with a threshold of <50 nmol/L most commonly used to define vitamin D deficiency [17]. Vitamin D deficiency is common in CKD stages 3–5, with a reported prevalence of over 40% [18, 19]. Observational studies have shown an association between vitamin D deficiency and a range of adverse outcomes in people with CKD including reduced bone mineral density (BMD), vascular calcification, left ventricular hypertrophy and mortality [20–24]. However observational cohort studies are hypothesis-generating and should not be considered confirmatory that associations of vitamin D deficiency are causative in nature. Randomized controlled trials (RCTs) are required for evidence-based management of vitamin D deficiency.

## EFFECTS OF VITAMIN D THERAPY ON MORTALITY AND CARDIOVASCULAR OUTCOMES

### Trial evidence

A 2017 meta-analysis identified 17 RCTs and 21 observational studies which examined the effect of vitamin D therapy on mortality in adults with CKD stages 1–5 [25]. In observational studies, vitamin D therapy was associated with a reduced risk of all-cause mortality [relative risk (RR) 0.62, 95% confidence interval (CI) 0.52 to 0.72]. The effect was consistent across various subgroups including CKD stages, size of study, routes of administration and types of vitamin D agent used. However, in the meta-analysis of RCTs, there was no significant difference in risk of all-cause mortality (RR 1.26, 95% CI 0.77 to 2.05).

This finding was similar to an updated 2023 meta-analysis that included 128 RCTs examining vitamin D therapy in CKD [2]. Of these, 26 placebo-controlled RCTs reported all-cause mortality and 10 reported a major adverse cardiovascular event (MACE) outcome. Overall, vitamin D therapy did not significantly reduce the risk of mortality (pooled RR 1.04, 95% CI 0.84 to 1.24) or MACE (pooled RR 0.94, 95% CI 0.62 to 1.42) [2]. Only two RCTs had MACE and all-mortality as primary and secondary outcomes rather than an adverse event (Table 1). Both trials were conducted in people with kidney failure on haemodialysis. One investigated the VDRA alfacalcidol and the other investigated the nutritional vitamin D supplement calcifediol.

**Table 1:** Overview of major RCTs examining the effect of vitamin D supplementation on bone and cardiovascular outcomes in the general population and people with CKD.

Study	*N*, participants	Intervention/follow-up	Outcome: fractures	Outcome: MACE	Outcome: all-cause mortality
General population					
WHI 2006 [62, 63]	36 282 post-menopausal women, aged 50–79 years	Calcium 1000 mg + vitamin D3 400 IU daily vs placebo 7 years	• Hip fractures: HR 0.88 (0.72 to 1.08)^a^ • Vertebral fractures: HR 0.90 (0.74–1.10) • Forearm/wrist fractures: HR 1.01 (0.90–1.14) • Total fractures: HR 0.96 (0.91–1.02)	• Composite MACE (MI, CV death, CABG or PCI): HR 1.08 (0.99–1.19) • Hospitalized HF: HR 0.96 (0.83–1.10) • Stroke: HR 0.95 (0.82–1.10)	HR 0.91 (0.83–1.01)
					
VITAL 2019 [64, 65]	25 871 older adults (men >50 years, women >55 years)	Vitamin D3 2000 IU daily vs placebo 5.3 years	• Total fractures: HR 0.98 (0.89–1.08) • Non-vertebral fractures: HR 0.97 (0.87–1.07) • Hip fractures HR 1.01 (0.70–1.47)	Composite MACE (MI, stroke or cardiovascular death): HR 0.97 (95% CI 0.85–1.12)^a^	HR 0.99 (0.87–1.12)
D-Health 2022 [30, 66, 67]	21 315 older adults, aged 60–84 years	Vitamin D3 60 000 IU monthly vs placebo 5 years	• Total fractures: HR 0.94 (0.84–1.06) • Non-vertebral fractures: HR 0.96 (0.85–1.08)• Major osteoporotic fractures: HR 1.00 (0.85–1.18)• Hip fractures: HR 1.11 (0.86–1.45)	Composite MACE (MI, stroke or coronary revascularization): HR 0.91 (0.81–1.01)	HR 1.04 (0.93–1.18)^a^
RECORD 2005 [68]	5292 older adults (age >70 years) with a low-trauma, osteoporotic fracture in past 10 years	2 × 2 factorial design: Vitamin D3 800 IU daily vs D3 800 IU plus calcium 1000 mg daily vs placebo 3.8 years	• Low-trauma fractures: HR 1.02 (0.88–1.19)^a^ • Any new fracture: HR 1.01 (0.88–1.17)	• Composite MACE (fatal and non-fatal HF, MI, stroke): HR 0.92 (0.80–1.08) • Composite fatal CVD (HF, MI and stroke): HR 0.87 (0.73–1.03)	HR 0.92 (0.80–1.05)
VIDA 2017 [69, 70]	5110 community-dwelling adults, aged 50–84 years 3.3 years	Vitamin D3 200 000 IU then 100 000 IU monthly vs placebo	Non-vertebral fractures: HR 1.19 (0.94–1.50)	• Incident CVD and death: HR 1.02 (0.87–1.20)^a^ • MI: HR 0.90 (0.54–1.50) • Heart failure: HR 1.19 (0.84–1.68) • Stroke: HR 0.95 (0.55–1.62)	HR 1.12 (0.79–1.58)
Vital D 2010 [71]	2256 community-dwelling women ≥70 years with high fracture risk	Vitamin D3 500 000 IU yearly vs placebo 2.96 years	• Any new fracture: RR 1.26 (1.00–1.59)^†^ • Non-vertebral fracture: RR 1.28 (1.00–1.65)	• Not reported	No difference between vitamin D (40/1131) and placebo group (47/1125)
CKD population					
J-DAVID 2018 [26]	976 haemodialysis patients with serum iPTH ≤180 pg/mL	Alfacalcidol 0.5 µg daily vs no treatment 4.0 years	No difference in fractures between alfacalcidol (9/488) and control (12/476) groups	• Composite MACE (fatal and non-fatal MI, HF hospitalizations, stroke, aortic dissection/rupture, amputation of lower limb due to ischaemia, sudden cardiac death; coronary revascularization; and leg artery revascularization): HR 1.25 (0.94–1.67)^a^	HR 1.12 (0.83–1.52)
					
Morrone 2022 [27]	284 haemodialysis patients (age ≥18 years) with serum PTH 2–9× ULN and 25(OH)D <30 ng/mL	Calcifediol 40 mcg thrice weekly after dialysis vs no treatment 2.0 years	Not reported	• Composite MACE (non-fatal MI, non-fatal stroke, all-cause death): HR 1.03 (0.63–1.67)^a^ • Cardiovascular death: HR 1.06 (0.41–2.74)	HR 1.11 (0.67–1.83)
PRIMO 2011 [28]	227 patients with eGFR 15–60 mL/min/1.73 m^2^, serum iPTH 50–300 pg/mL	Paricalcitol 2 µg daily vs placebo 48 weeks	No difference in fractures between paricalcitol (1/115) and placebo group (2/112)	Fewer hospitalizations for CVD events in paricalcitol group (1/115 vs 7/112, *P *= .03)	No deaths during study period
FLUID 2022 [44]	65 peritoneal dialysis patients (age ≥18 years)	Cholecalciferol 50 000 IU weekly for 8 weeks followed by 10 000 IU weekly for 44 weeks vs placebo 52 weeks	No difference in fractures between vitamin D (1/43) or placebo groups (2/31), *P *= .5	• Fewer cardiovascular deaths in vitamin D group (1/34 vs 6/31, *P *= .03) • No difference in MACE (MI, HF hospitalization, coronary revascularization, stroke, limb amputation or peripheral revascularization): 5/34 vs 7/31 (*P *= .4)	Fewer deaths in vitamin D group (4/34 vs 12/31, *P *= .004)
OPERA 2014 [29]	60 patients with non-dialysis CKD (eGFR <60 mL/min/1.73 m^2^), serum iPTH >55 pg/mL	Paricalcitol 1 µg daily (if iPTH <500 pg/mL) or 2 µg daily (if iPTH ≥500 pg/mL) vs placebo 52 weeks	Not reported	• Fewer cardiovascular events (0/30 vs 6/30) in paricalcitol group • Fewer cardiovascular-related hospitalizations (0/30 vs 5/30) in paricalcitol group	No deaths during study period

^a^Primary study outcome.

iPTH, intact PTH; ULN, upper limit of normal; IU, international units; MI, myocardial infarction; CV, cardiovascular; CVD, cardiovascular disease; CABG, coronary artery bypass graft; PCI, percutaneous coronary intervention; HF, heart failure; VITAL, Vitamin D and Omega-3 trial; WHI, Women's Health Initiative; RECORD, Randomised Evaluation of Calcium or Vitamin D; VIDA, Vitamin D Assessment.

The first was the J-DAVID (Japan Dialysis Active Vitamin D) trial, which randomized 976 people on haemodialysis to oral alfacalcidol 0.5 µg daily or standard therapy [26]. Participants were recruited from 207 dialysis units in Japan. At 48 months of follow-up, there was no significant difference in risk of all-cause mortality [hazard ratio (HR) 1.12, 95% CI 0.83 to 1.52] or MACE (HR 1.25, 95% CI 0.94 to 1.67) between the two treatment groups.

The second trial by Morrone *et al.* published in 2022 randomized 284 people on haemodialysis with hyperparathyroidism and vitamin D insufficiency [25(OH)D <30 ng/mL] to oral calcifediol 40 µg thrice weekly or standard care [27]. Participants were recruited from 28 dialysis units around Italy. Of note, 60% of participants were also on an VDRA (calcitriol or paricalcitol) at baseline although there was no difference in rate of use between the two treatment groups during the study. At 24 months of follow-up, there was no difference in all-cause mortality (HR 1.11, 95% CI 0.67 to 1.83), cardiovascular mortality (HR 1.06, 95% CI 0.41 to 2.74) or MACE (HR 1.03, 95% CI 0.63 to 1.67).

Compared with the J-DAVID cohort, participants in the Morrone *et al.* trial had a higher prevalence of cardiovascular disease (52.5% versus 25%), more severe hyperparathyroidism (baseline median serum PTH 240 vs 85.6 pg/mL) and shorter dialysis vintage (mean 2.9 vs 5.5 years).

### Summary

Despite the findings from observational studies that vitamin D deficiency is associated with increased mortality risk in CKD patients, there is now moderate certainty evidence that vitamin D therapy does not reduce risk of all-cause mortality in people with kidney failure on haemodialysis compared with standard care. However, there have been no RCTs specifically examining the effect of vitamin D therapy on mortality or cardiovascular endpoints in people with non-dialysis CKD. As shown in Table 1, two of the larger vitamin D trials conducted in this population reported no deaths during their study periods [28, 29]. Sample sizes were 227 and 60, and study durations were 48 and 52 weeks, respectively. In comparison, the largest trial conducted assessing the effect of vitamin D therapy on mortality in the general population was the D-Health trial which randomized 21 315 older adults aged 60 years or older to cholecalciferol 60 000 IU monthly or placebo for 5 years [30]. Given the lower mortality and cardiovascular risk in the non-dialysis population, any future studies would need to be of larger size and longer duration to have adequate statistical power to detect an effect on cardiovascular endpoints and all-cause mortality.

## EFFECTS OF VITAMIN D THERAPY ON INTERMEDIATE CARDIOVASCULAR OUTCOMES

### Endothelial function and arterial stiffness

Endothelial dysfunction in CKD results from accumulation of uraemic toxins, reduced nitric oxide production, oxidative stress and increased systemic inflammation [31, 32]. Impaired endothelium-dependent vasodilation and endothelial inflammation have been linked to hypertension and atherosclerosis [33]. Another marker of vascular function is arterial stiffness, which has been associated with a range of adverse outcomes in CKD patients including proteinuria, heart failure, CKD progression and death [34]. Reduced flow-mediated dilation (FMD), a marker of abnormal vasodilatory function, and increased pulse wave velocity (PWV), a measure of arterial stiffness, are both commonly seen in people with CKD and have been linked to vitamin D status [4, 15, 35].

In one clinical trial, 120 people with stage 3–4 CKD and vitamin D deficiency were randomized to high-dose cholecalciferol 300 000 IU or matching placebo at baseline and 8 weeks [36]. At 16 weeks, the cholecalciferol group had a significantly higher FMD compared with placebo, with a between-group difference of 5.49%. The Paricalcitol and Endothelial Function in Chronic Kidney Disease (PENNY) Trial randomized 88 people with CKD stage 3–4 to paricalcitol or placebo [37]. After 12 weeks, FMD was significantly higher in the paricalcitol group, with a between-group difference of 1.8%. However, in a head-to-head trial between cholecalciferol and calcitriol in 128 people with CKD stage 3b–4 and vitamin D deficiency, there was no significant difference in change in FMD between the two groups after 6 months [38].

In a Canadian trial of 119 people with CKD stage 3b–4, participants were randomized to placebo, calcifediol or calcitriol [39]. After 6 months, there was a significant reduction in PWV in the calcifediol group compared with placebo, with a between-group difference of −2.1 m/s. Notably, baseline PWV values were not balanced across the three groups. The placebo group had the lowest mean PWV (mean 10.7 m/s) and the calcifediol had the highest (12.1 m/s). After adjusting for baseline PWV, the difference was no longer statistically significant. Similarly, another smaller trial of 36 people with CKD randomized to placebo, 1 or 2 μg of paricalcitol, found no change in PWV in any of the groups after 3 months [40].

### Vascular calcification

A 2022 meta-analysis identified six trials (total 289 participants) examining the effect of vitamin D therapy on vascular calcification in people with CKD, two of which were non-randomized [41]. Half evaluated cholecalciferol and the other half evaluated calcitriol. Sample size ranged from 36 to 76 participants, and study duration ranged from 6 to 60 months. There was considerable variation in how vascular calcification was assessed, from X-rays of the hands and abdominal aorta to computed tomography (CT) of coronary arteries. None of the trials demonstrated any benefit with vitamin D therapy on vascular calcification in any of the anatomical sites studied.

The largest trial was published almost 40 years ago in 1986 by Baker *et al.*, where 76 people on haemodialysis were randomized to oral calcitriol or placebo for 60 months [42]. Vascular calcification was a secondary endpoint and assessed using X-rays of the hands, feet, pelvis and coronary arteries. The most recent placebo-controlled trial was by Samaan *et al.* (2019), in which 47 patients with a creatinine clearance 15–60 mL/min and a 25(OH)D level of 16–29 ng/mL were randomized to cholecalciferol or placebo [43]. Coronary artery calcium score was obtained by CT at baseline and 18 months. In the multivariate analysis, there was no difference in change in vascular calcification between placebo and cholecalciferol groups.

### Left ventricular mass

A 2023 meta-analysis identified seven placebo-controlled RCTs examining left ventricular mass in adults with CKD, all of which showed no significant change with vitamin D therapy [2]. Vitamin D agents investigated included cholecalciferol, ergocalciferol, paricalcitol and alfacalcidol. Left ventricular mass was the primary outcome in six of these trials and follow-up duration ranged from 6 months to 1 year [28, 29, 44–47].

The largest of these was the Paricalcitol Capsule Benefits in Renal Failure–Induced Cardiac Morbidity (PRIMO) Trial, which randomized 227 patients with CKD stages 3–4 and mild to moderate left ventricular hypertrophy to oral paricalcitol 2 μg daily or matching placebo [28]. The primary outcome was left ventricular mass index (LVMI) assessed using cardiac magnetic resonance imaging (MRI) and secondary outcomes were various measures of diastolic function on transthoracic echocardiogram. This was a multinational trial, with a predominantly white (74%) study population. At 48 weeks, no significant difference was reported between treatment groups in either the primary or secondary outcomes.

The OPERA trial randomized 60 people with stages 3–5 non-dialysis CKD and left ventricular hypertrophy to paricalcitol 1–2 μg daily (depending on serum PTH) or matching placebo [29]. Participants were recruited from a single centre in Hong Kong and predominantly Chinese. Similar to the PRIMO trial, the primary outcome was LVMI assessed by cardiac MRI and secondary outcomes were other measures of cardiac function on MRI and echocardiography. At 52 weeks, there was no difference in primary or secondary outcomes between groups. Compared with the PRIMO cohort, OPERA trial participants had higher baseline LVMI indexed to height^2.7^ (mean 38.6 versus 23.7 g/m^2^) and more severe CKD [median estimated glomerular filtration rate (eGFR) 21.8 vs 33.5 mL/min/1.73 m^2^].

Two trials have been conducted in people on dialysis and examined use of high-dose cholecalciferol. The first is the Fluid Management Using Bio-Impedance in Peritoneal Dialysis (FLUID) trial which randomized 65 people on peritoneal dialysis to cholecalciferol 50 000 IU weekly for 8 weeks followed by 10 000 IU weekly for 44 weeks or placebo [44]. At 52 weeks, there was no difference in LVMI as assessed by cardiac MRI between groups. The second by Mose *et al.* (2014) randomized 64 people on haemodialysis to cholecalciferol 3000 IU daily or standard care [45]. At 6 months, there was no difference in LVMI as assessed by transthoracic echocardiography.

### Summary

Key findings of clinical trials assessing effects of vitamin D therapy on intermediate cardiovascular measures are summarized in Table 2. There are no high-quality RCTs evaluating outcomes such as FMD, PWV and vascular calcification in people with CKD. Most findings have been negative but certainty of evidence is low and it is difficult to draw definitive conclusions. Given the increased risk of hypercalcaemia and hyperphosphataemia associated with VDRA use, there is theoretical concern that VDRAs could also worsen vascular calcification. Further placebo-controlled studies of nutritional vitamin D and VDRAs are needed to assess the effect of vitamin D therapy on vascular calcification and arterial stiffness in both dialysis and non-dialysis CKD patients. Use of standardized methods for quantifying vascular calcification, such as a coronary artery calcium score from CT and a Kauppila score for abdominal aortic calcification based on lateral lumbar spine plain radiography, would also be important to evaluate this intermediate cardiovascular outcome [48].

**Table 2:** Summary of key data published for vitamin D therapy in intermediate cardiovascular outcomes in CKD.

Outcome	Key facts
Endothelial function	• Vitamin D therapy may improve FMD compared with placebo in patients with stage 3–4 CKD [36, 37]
Arterial stiffness	• Existing trials suggest vitamin D has no effect on PWV in non-dialysis CKD patients [36, 40, 45, 72]
Vascular calcification	• No significant effect on vascular calcification in non-dialysis CKD patients [42, 43, 73, 74]
LVMI	• Vitamin D has no significant effect on LVMI in stage 3–5D CKD patients [28, 29, 44–47]

In contrast, there have been more high-quality trials performed on the effect of vitamin D therapy on left ventricular hypertrophy in people with CKD. Findings from the PRIMO and OPERA trials suggest that paricalcitol does not reduce LVMI compared with placebo in people with non-dialysis CKD. Evidence in people on dialysis is less clear, although findings from existing trials suggest cholecalciferol does not reduce LVMI compared with placebo.

## EFFECTS OF VITAMIN D THERAPY ON BONE HEALTH

### Fractures

Given the well-known role of vitamin D in bone metabolism, it is not surprising that multiple large RCTs have been conducted on older adults in the general population examining the effect of vitamin D therapy on fracture risk (Table 1). The sample sizes are in the thousands with follow-up of up to 7 years. All have investigated cholecalciferol, which is the most commonly prescribed agent for vitamin D deficiency in the general population. Interestingly, none of the trials has demonstrated any significant benefit in reducing fracture risk.

In contrast, there have been no RCTs on vitamin D therapy in CKD specifically examining fracture as the primary outcome. A 2023 meta-analysis identified eight RCTs comparing vitamin D to placebo which reported fractures [2]. There was a trend towards reduced fracture risk with VDRAs but the result was not statistically significant. The largest of the included RCTs was the J-DAVID trial, where fractures were examined as a serious adverse event rather than a study outcome [26]. There were 9 events in the intervention group compared with 12 in the control group [26]. The PRIMO trial also reported fractures as an adverse event [28]. There was no significant difference in the rate of fractures: 1/115 in the paricalcitol group as compared with 2/112 in the placebo group.

### Bone mineral density

Several observational studies have reported an association between 25(OH)D deficiency and low BMD in people with non-dialysis CKD stage 3–5 [24, 49]. In a *post hoc* analysis of the Vitamin D, Calcium, Lyon Study II (DECALYOS II) study, which randomized 610 elderly women to cholecalciferol and calcium supplementation or placebo for 2 years, participants overall experienced a decrease in BMD over time with active treatment [50]. However, in a subset of 100 women on cholecalciferol and calcium therapy with an eGFR <45 mL/min, the rate of BMD loss appeared to be slower compared with those receiving placebo, although it is important to note that women with a serum creatinine >150 µmol/L were excluded from the trial. Two smaller trials in people with non-dialysis CKD found that calcitriol and alfacalcidol, both VDRAs, increased BMD compared with placebo over 12–18 months follow up [51, 52]. However, study numbers were small with fewer than 40 participants in each trial.

In comparison, a 2014 systematic review identified 23 RCTs (total 4082 participants, mean duration 23.5 months) assessing effects of nutritional vitamin D on BMD in the general population [53]. Overall, vitamin D resulted in a small but statistically significant increase in femoral neck BMD with a weighted mean difference (WMD) of 0.8% (95% CI 0.2 to 1.4, *P *= .005), but not in other areas including the lumbar spine, total hip, total body or forearm. Studies with positive outcomes tended to have lower baseline 25(OH)D levels and were of longer duration (12 months or more). All positive trials were conducted in women.

Eldecalcitol, a VDRA, has also been studied in the general population for treatment of osteoporosis and has been approved for this indication in Japan since 2013 [54]. A 2022 systematic review and meta-analysis identified eight RCTs (total 2368 participants) comparing the effect of eldecalcitol versus another comparator (alfacalcidol, bisophosphate or placebo) [55]. Seven trials were performed in Japan and one in China. Eldecalcitol significantly increased femoral neck BMD (WMD 0.92, 95% CI 0.24 to 1.60), but had no effect on lumbar spine or hip BMD. Perhaps more importantly, eldecalcitol significantly reduced the risk of osteoporotic fractures (RR 0.70, 95% CI 0.55 to 0.88) and vertebral fractures (RR 0.74, 95% CI 0.55 to 0.98).

### Renal osteodystrophy

In a multicentre trial of 176 people with CKD with a creatinine clearance of 15–50 mL/min, participants were randomized to oral alfacalcidol or placebo for 2 years [56]. At baseline, 132 patients had abnormal bone histology, including 98 with osteitis fibrosa. Among people with abnormal bone histology at baseline, bone disease had resolved in 42% of those who received alfacalcidol compared with 4% on placebo at the end of the study.

However, there has long been concern that over-suppression of PTH with vitamin D can lead to development of adynamic bone disease, especially in people on dialysis [57]. In a study of 14 paediatric patients on peritoneal dialysis, intermittent calcitriol thrice weekly for 12 months resulted in complete resolution of osteitis fibrosa in 10 of the 11 patients with this condition at baseline, although 6 patients developed adynamic bone lesions [58].

Over-suppression of PTH could be avoided by monitoring and aiming for higher PTH targets during treatment, as shown by a small prospective study of 43 African-American people on haemodialysis [59]. Participants were divided in two groups and received active vitamin D therapy according to two separate protocols aiming for different PTH targets. After 3 years, 7/22 (32%) of the group with the lower PTH target were found to have adynamic bone disease on bone biopsy as opposed to 0/21 in the higher PTH target group. However, no bone biopsies were performed at baseline and the study was not randomized.

### Summary

Key findings on the effect of vitamin D therapy on bone outcomes in people with CKD are summarized in Table 3. Overall, there is very limited data and much larger trials are needed of sufficient duration, similar those conducted in the general population, to assess the effect of vitamin D therapy on fractures in both dialysis and non-dialysis CKD patients. Existing data suggest that VDRAs have the potential to increase BMD, improve renal osteodystrophy and reduce fracture risk in CKD. In people on dialysis, it would be important to monitor PTH levels during treatment to avoid over-suppression and development of adynamic bone disease.

**Table 3:** Summary of key data published for vitamin D therapy in bone health in CKD.

Outcome	Key facts
BMD	• Treatment with cholecalciferol and calcium may attenuate BMD loss in elderly women with stage 3b–4 CKD [50]
	• VDRA therapy may increase BMD in patients with non-dialysis CKD compared with placebo [51, 52]
Renal osteodystrophy	• VDRA therapy may improve high bone turnover disease (osteitis fibrosa) in some patients with stage 3–5D CKD, but at the risk of developing adynamic bone disease [56, 58]
	• Aiming for a lower PTH target may reduce the risk of adynamic bone disease in haemodialysis patients treated with a VDRA [59]
Fractures	• No RCTs with fracture as primary outcome
	• Uncertain whether vitamin D reduces fracture risk in CKD [2]

## DISCUSSION

Many observational studies have demonstrated an association between vitamin D deficiency and various adverse outcomes including fractures, falls, vascular dysfunction, cardiovascular risk and mortality in both the general and CKD populations. However, inherent methodological limitations of observational studies including confounding, selection bias and imprecise exposure quantification mean that causal inferences cannot be made. Serum 25(OH)D levels are used to define vitamin D deficiency, but this may not be the most accurate reflection of vitamin D status particularly in people with CKD where there is reduced conversion of 25(OH)D to 1,25(OH)_2_D. Other factors can affect 25(OH)D levels such as nutrition and sunlight exposure. 25(OH)D could be a more general marker of overall health, which is difficult to adjust for. Furthermore, the lack of standardization in 25(OH)D assays and variable definitions of what constitutes 25(OH)D deficiency can make findings of observational studies more difficult to interpret [17, 60].

The majority of RCTs on vitamin D therapy which have been performed in the CKD population have focused mainly on surrogate biochemical rather than clinical outcomes. Clinical endpoints are often reported as an adverse effect rather than a main outcome of the trial. In the case of calcimimetics for treatment of secondary hyperparathyroidism, biochemical effects of PTH suppression did not translate into improvements in all-cause or cardiovascular mortality [61]. Sample sizes of the trials in people with CKD are also significantly smaller than trials in the general population, some of which have included over 20 000 participants. This fact was highlighted in a recent meta-analysis which identified 128 RCTs on vitamin D therapy in adults with CKD [2]. The majority of studies were of low to moderate quality, with small sample sizes and short follow-up duration. This makes it difficult to know whether the lack of positive findings was due to a true absence of effect or poor trial design.

## CONCLUSION AND FUTURE DIRECTIONS

Vitamin D plays an essential role in calcium and phosphate homeostasis, as well as bone formation and mineralization. Vitamin D metabolism is altered as kidney function declines, contributing to the pathogenesis of CKD-MBD. The result is not only significant bone disease, but also increased cardiovascular and mortality risk through endothelial dysfunction, vascular calcification and arterial stiffness.

Vitamin D therapy, both active and nutritional, is widely used as treatment for CKD-MBD. Evidence is mainly for intermediate outcomes such as lowering PTH, but existing trials have not shown any significant benefit for clinical outcomes. Future trials should focus on the role of activated vitamin D compounds on clinical outcomes, particularly fractures and bone health (Table 4). Current trial evidence in both the general and CKD populations suggests that VDRAs may improve BMD and reduce fracture risk, and there is biological plausibility given the established role of vitamin D in bone and mineral metabolism and known effects of kidney impairment on reduced calcitriol synthesis. Investigating whether VDRAs improve or potentially worsen vascular calcification is important, given increased risk of hypercalcaemia with these agents. Future trials need to be powered appropriately, and be of adequate size and duration to detect these outcomes, with a particular focus more specifically on selective populations such as those with secondary hyperparathyroidism and/or vitamin D deficiency.

**Table 4:** Suggestions for areas of future research.

Suggestions for future research
• Future studies need to be of adequate size and duration to provide the statistical power to examine the effect of vitamin D therapy in CKD
• Identify optimal PTH target in non-dialysis CKD patients
• Potential areas of research for VDRAs in CKD:
o Fractures and BMD in both dialysis and non-dialysis population
o Mortality and cardiovascular endpoints in non-dialysis CKD population
o Vascular calcification and arterial stiffness in both dialysis and non-dialysis CKD population

Based on current evidence, we do not recommend routine use of vitamin D supplementation in people with CKD stages 3–5, although in those with 25(OH)D deficiency there may be some rationale for replacement as benefit may outweigh harm and physiologically this may benefit bone health. VDRAs can be considered for treatment of severe hyperparathyroidism (which is persistent and progressive), although an optimal PTH target for people with non-dialysis CKD has not been established. There is currently no convincing evidence that nutritional vitamin D supplementation or active vitamin D therapy reduces cardiovascular disease or mortality risk in people with CKD.

## Data Availability

No new data were generated or analysed in support of this research.
